# Enhanced HIV SOSIP Envelope yields in plants through transient co-expression of peptidyl-prolyl isomerase B and calreticulin chaperones and ER targeting

**DOI:** 10.1038/s41598-022-14075-3

**Published:** 2022-06-15

**Authors:** Yvonne J. Rosenberg, Xiaoming Jiang, Jonathan P. Lees, Lori A. Urban, Lingjun Mao, Markus Sack

**Affiliations:** 1 PlantVax Corporation, Rockville, MD USA; 2 Pro-SPR GmbH, Alsdorf, Germany

**Keywords:** Biological techniques, Biotechnology, Drug discovery, Plant sciences

## Abstract

High yield production of recombinant HIV SOSIP envelope (Env) trimers has proven elusive as numerous disulfide bonds, proteolytic cleavage and extensive glycosylation pose high demands on the host cell machinery and stress imposed by accumulation of misfolded proteins may ultimately lead to cellular toxicity. The present study utilized the *Nicotiana benthamiana/p19 (N.b./p19*) transient plant system to assess co-expression of two ER master regulators and 5 chaperones, crucial in the folding process, to enhance yields of three Env SOSIPs, single chain *BG505 SOSIP.664 gp140, CH505TF.6R.SOSIP.664.v4.1* and *CH848-10.17-DT9*. Phenotypic changes in leaves induced by SOSIP expression were employed to rapidly identify chaperone-assisted improvement in health and expression. Up to 15-fold increases were obtained by co-infiltration of peptidylprolvl isomerase (PPI) and calreticulin (CRT) which were further enhanced by addition of the ER-retrieval KDEL tags to the SOSIP genes; levels depending on individual SOSIP type, day of harvest and chaperone gene dosage. Results are consistent with reducing SOSIP misfolding and cellular stress due to increased exposure to the plant host cell’s calnexin/calreticulin network and accelerating the rate-limiting cis–trans isomerization of Xaa-Pro peptide bonds respectively. Plant transient co-expression facilitates rapid identification of host cell factors and will be translatable to other complex glycoproteins and mammalian expression systems.

## Introduction

The glycoprotein envelope spike on the surface of HIV virions is the sole target for neutralizing antibodies and thus recombinant envelope proteins have been the focus of HIV vaccine design. The critical antigenic structures that cover most of the envelope surface, include both glycans and peptides, and have been identified by highly mutated potent HIV monoclonal antibodies derived by cloning B cells from infected individuals^1,2^ and shown to prevent infection in animal models and suppress HIV viremia in humans^3–8^ as a result of their anti-viral activity against a wide spectrum of viruses by targeting relatively conserved regions on the surface HIV envelope trimer^9–11^. These broadly neutralizing antibodies (bnAbs) are designated by their recognition of different sites of vulnerability on the envelope e.g. V1/V2, V3 + glycans, MPERS, the gp120-gp41 interface, and the CD4-binding site with some overlap between sites^9–12^. More recently, rational vaccine designs have targeted the latter CD4 binding sites (CD4bs) and the gp41 elements proximal to the furin cleavage site, representing less occluded gaps in the Env glycan shield.

Despite best efforts, previous HIV vaccine approaches have failed to induce antibodies capable of neutralizing heterologous Tier-2 primary viruses, until the recent development of stabilized Env trimers that structurally represent the native virion-bound Env spike. Several e.g. the cleavage independent, native flexibly linked (NFL) Clade C HIV-1 envelope glycoprotein Env trimers with targeted N-glycan deletions^13^, the soluble single chain BG505.SOSIP gp140 trimer with a flexible linker to replace the cleavage site^14^ and the cleaved Clade A BG505.664 gp140 SOSIP pioneered by Sanders and Moore^15,16^ have shown promise in terms of immunogenicity in macaques and rabbits^13,17–20^;the latter representing the starting point for strategies aimed at improving vaccine design and increased efficacy. Cleaved SOSIP trimers are highly glycosylated (75–90 glycans/trimer), metastable complexes of three gp120 and three gp41 ectodomain subunits held together by non-covalent interactions and characterized by (i) an engineered intersubunit disulfide bond (SOS) between gp120 and the gp41 ectodomain, (ii) a stabilizing substitution (I559P) to maintain the SOSIPs in their pre-fusion form, and (iii) truncation at gp41 residue 664 to remove the hydrophobic transmembrane region which exacerbates fragility and solubility^15,16^. Many sequence and structural changes to the SOSIP trimers have been made to (i) stabilize the protein (ii) expose as many bnAb epitopes as possible^21^ while (iii) occluding those for non-neutralizing antibodies^12^. In addition, several other HIV Env proteins from diverse Clade subtypes have now also been expressed as SOSIPs^17^ and a BG505 SHIV has been studied in macaques^22^. However, compared to other recombinant proteins such as monoclonal antibodies which are made at g/L levels in CHO or HEK29 cell bioreactors, complex HIV SOSIP envelope glycoproteins have been generally more difficult to produce in most expression systems at high yields. Clearly the SOSIP structure imposes a higher demand on the host cell machinery in terms of folding requirements and assistance by ER chaperones. Transient expression in Agrobacterium-mediated plant-based systems^23,24^ provide unique opportunities for the rapid manipulation of host cell factors to identify the bottle neck for high production.

In this context, the *Nicotiana benthamiana* (*Nb*) plant-expression system, which is fast and scalable with lower requirements for the sophisticated equipment required for mammalian cell-based manufacturing has become increasingly popular for the production of human biologics (vaccines, enzymes, growth factors) and monoclonal antibodies^25–28^. These systems are especially important for time critical applications e.g. the rapid production of anti-Ebola antibodies^29^, anti-flu mAbs and flu subunit vaccines and more recently, development of vaccine and antibodies against the new COVID-19 pandemic strains. Many plant-derived recombinant proteins have progressed to clinical testing (e.g. lactoferrin (Ventria)^30^, COVID-19 vaccines (Medicago/GSP^31^), while plant-cell derived recombinant glucocerebrosidase (Protalix Biotherapeutics) has received FDA regulatory approval as a human treatment for Gaucher disease (www.protalix.com).

Importantly, plant-produced proteins are essentially indistinguishable from those in animals with respect to protein synthesis, secretion and chaperone-assisted protein folding, together with the post-translational modification processes (e.g. signal peptide cleavage, disulfide bond formation and the *early stages* of N-linked glycosylation). Plant systems also provide excellent models for controlling subcellular trafficking of proteins to achieve further differential glycosylation profiles when required e.g. proteins targeted to the ER by a C-terminal SEKDEL retrieval signal and which carry only high mannose N-glycans. In this context, the predominance of oligomannose type (OMT) N-glycans on plant-derived proteins may permit production of recombinant HIV Env molecules that more closely resemble native trimers^32,33^.

Several forms of HIV Env have previously been produced in the *Nicotiana* species plant expression system. The 89.6P gp140 Env was expressed in both transgenic SR1 *Nicotiana tabacum* plants^26^ while both the KDEL-tagged and unmodified versions have been expressed in *N. benthamiana* at ~ 30 mg/kg levels. More recently, transient expression in *N. benthamiana* of trimeric HIV Env gp140 SOSIP.664 antigens based on two rationally selected virus isolates CAP256 and Du151 has been demonstrated with increased expression of CAP256 as well as other viral glycoproteins by manipulation of the chaperone folding machinery^34,35^. Consistent with SOSIP-induced toxicity, plant expression of SOSIP trimers has been associated with considerable leaf pathology and wilting, which has been reported to be associated with ER stress caused by accumulation of misfolded of viral and bacterial glycoproteins^35–37^.

Regardless of the expression system used, eukaryotic cells have in place various quality control systems to support folding of nascent polypeptide chains and to identify and degrade misfolded proteins in the endoplasmic reticulum associated degradation (ERAD)^38^. Build-up of unfolded proteins causes ER stress and triggers strong cellular responses, the unfolded protein response (UPR)^39^ that can eventually trigger cell cycle arrest and apoptosis. When recombinant genes are overexpressed, ER stress can be caused by consumption of host cell factors that are not available for endogenous proteins, and which in turn aggregate and are unavailable to sustain cellular homeostasis. This process is particularly important in plants because their sessile nature commands adaptation for survival rather than escape e.g. from abiotic stress. As such, plants make special use of the UPR, and evidence indicates that the master regulator and transcription factor bZIP-60-s and downstream effectors of the UPR have distinct roles in mediating cellular processes that affect organism growth and development as well as stress responses. It should be noted that HIV infection modulates the UPR in humans to enhance its own replication and secure infection success, while antiretroviral therapy can lead to activation of the unfolded protein response^40–43^.

This present study has focused on the UPR, specifically the Ire and ATF6 pathways, by co-expressing the *N. benthamiana* homologs of both the activated transcription factors/ master regulators and key ER chaperones^44–46^ (collectively referred to as ER stress modulators) listed below to assess their ability to enhance expression of three HIV SOSIP Envs.

1. *bZIP60-s* results from alternative splicing of bZIP60-u by Ire1 due to consumption of BiP by unfolded proteins and is the master transcription factor that upon trafficking to the nucleus induces expression of the Ire1 pathway of the UPR.
2. *bZIP28* is the functional equivalent of mammalian ATF6 and like Ire1 interacts with and is ER-retained by BiP under non-stress conditions.
3. *Protein disulfide isomerase* (PDI) Erp57, is a multi-functional protein that facilitates the formation of correct disulfide bonds between cysteine residues during the early stages of protein folding in the endoplasmic reticulum.
4. *Peptidyl-prolyl cis–trans isomerase* B (PPI-B, also known as CypB) is a highly conserved enzyme that catalyzes the *cis–trans* isomerization of proline imidic peptide bonds. PPI’s are vital for the folding of many proteins since proline *cis–trans* isomerization often is the rate limiting step in protein folding. PPI-B interacts with other ER chaperones to form foldase complexes and is significantly upregulated in the nuclei of HIV-infected monocyte-derived macrophages^47^. PPIs have been shown previously to improve refolding of gp41 expressed in *E. coli*^48^.
5. *Binding immunoglobulin protein* (BiP) also known as heat shock 70 kDa protein 5 (HSPA5) is a molecular chaperone encoded by the *HSPA5* gene in humans^49^. BiP is located in the ER lumen where it binds to newly synthesized proteins as they are translocated during translation, and maintains them in a state competent for subsequent folding and oligomerization.
6. *Calnexin* (CNX) and *calreticulin* (CRT) are calcium binding lectins recognizing GlcNAc2Man9Glc1 and function as molecular chaperones to assist in the folding and subunit assembly of the majority of Asn-linked glycoproteins. A concerted action between CNX/CRT, glucosidase II and UDP-glucose:glycoprotein glucosyltransferase (UGGT1) utilizes the terminal glucose residue as an indicator for incompletely folded glycoproteins^45^. Furthermore, it has been shown that postponed cleavage of the native gp160 signal peptide increases folding efficiency^50^ further emphasizing the delicate requirements of HIV Envs on the host cell machinery.

The findings demonstrate the ability of *N.benthamiana* ER stress regulators to mediate enhanced expression of three rationally designed HIV SOSIP Env trimers: (i) a soluble, single chain BG505 SOSIP.664 gp140 (scBG505) cleavage independent SOSIP (Sub-type A) based on the WT BG505^14^ with a 15 aa Gly-Ser linker (ii) CH505TF.6R. SOSIP.664.v4.1 SOSIP (CH505): a Clade C T/F virus with the BG505 gp41 which binds to the anti-CD4 CH103 bnAb unmutated common ancestor (UCA)^51^ with two mutations N279K and G458Y to render it susceptible to neutralization by the CH235 UCA and (iii) CH848.3.D0949.10.17CHIM.6R.SOSIP.664V4.1 (CH848*)* lacking N133 and N137 N-glycosylation sites permitting targeting and neutralization by the V3 bnAb DH270 UCA^52^.

## Results

### ELISA to detect SOSIP timers

To assess expression levels of SOSIP Env trimers in plants, a sandwich ELISA was developed using plant-derived trimer-specific PGT145 as a capture Ab and biotinylated plant 2G12 for detection. Importantly, PGT145 only recognizes a quaternary epitope at the apex of the Env trimer and does not bind to monomers. The long anionic HCDR3 of PGT145 has been shown to penetrate between glycans at the trimer threefold axis to contact peptide residues from all three Env protomers and accounts for its highly trimer-specific nature^53^.

**Figure 1:**
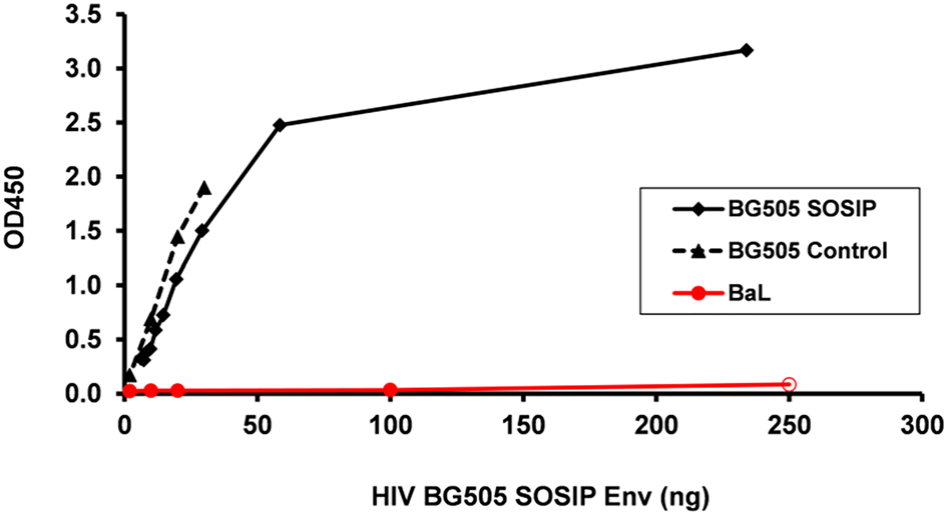
ELISA assay for the detection of plant derived HIV BG505 SOSIP Env trimers. Wells were coated with PGT145 and binding of samples (plant-BG505: black diamonds with solid line; CHO-BG505: black triangles with dashes line; monomeric BaLgp120: red circles with solid line) was revealed by biotinylated 2G12.

Figure 1 compares the binding of purified plant-derived scBG505 with a CHO-derived BG505 control and a monomeric BaLgp120 which is not recognized by PGT145. The results demonstrate recognition by PGT145 of both plant and CHO-BG505 molecules and a lack of binding to monomeric BaLgp120. The reactivity of plant BG505 SOSIP and CHO-derived BG505 Control molecules is very similar as shown by a slope-ratio of *m*_(BG505 SOSIP)_/*m*_(BG505 Control)_ = 0.859 (i.e. the ELISA reactivity differs by 14.1%) determined in the linear range of the sandwich ELISA.

### Toxicity of HIV SOSIP Env in Nicotiana species

**Figure 2:**
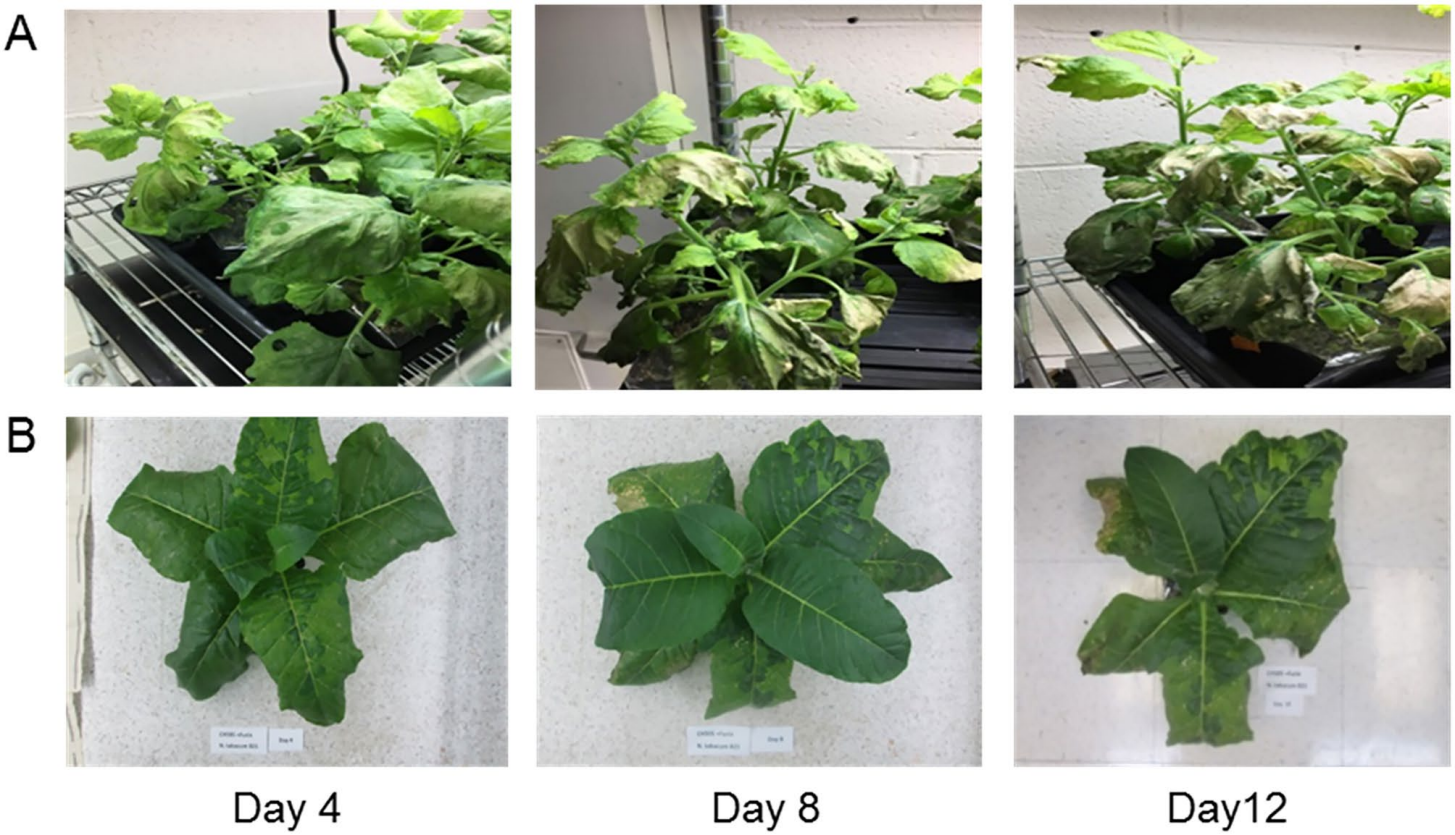
Phenotypic screening for SOSIP transgene overexpression induced stress in *Nicotiana* plants following infiltration with CH505 SOSIP genes. (**A**) *N. benthamiana* and (**B**) *N. tabacum* plants. Note: Holes in leaves represent areas where a leaf sample was taken. Upper leaves were removed from the *N. tabacum* plant to get a better view on the lower infiltrated leaves at days 4, 8 and 12.

Unlike most glycoproteins produced using plant expression systems, production of HIV Env by *Agrobacterium*-mediated co-transfection resulted in rapid wilting, browning and fragility of *N. benthamiana* leaves by ~ day 6; toxicity likely associated with protein misfolding and ER stress. Figure 2A shows the leaf pathology at 4-, 8- and 12-days post-infiltration (dpi) with genes for CH505 SOSIP (OD600 = 0.2), furin and p19 at a 3:1:0.6 ratio. A comparison of different SOSIP gene dosages, ranging from OD600 = 0.1 to 0.5, indicated that OD600 ~ 0.35 was optimal in terms of expression. Interestingly, infiltration of small *N.tabacum* (field tobacco) plants at OD600 = 0.8 showed no toxicity by 12 dpi (Fig. 2B); unless very high ODs of 1.5 were used. Thus, initial phenotypic screening and dot blot analysis (not shown) could provide a simple and rapid means for defining conditions and the ODs required for examining chaperone-enhanced expression before more time consuming assays were employed.

### Toxicity of individual ER chaperones in the absence of SOSIP *Env*

**Figure 3:**
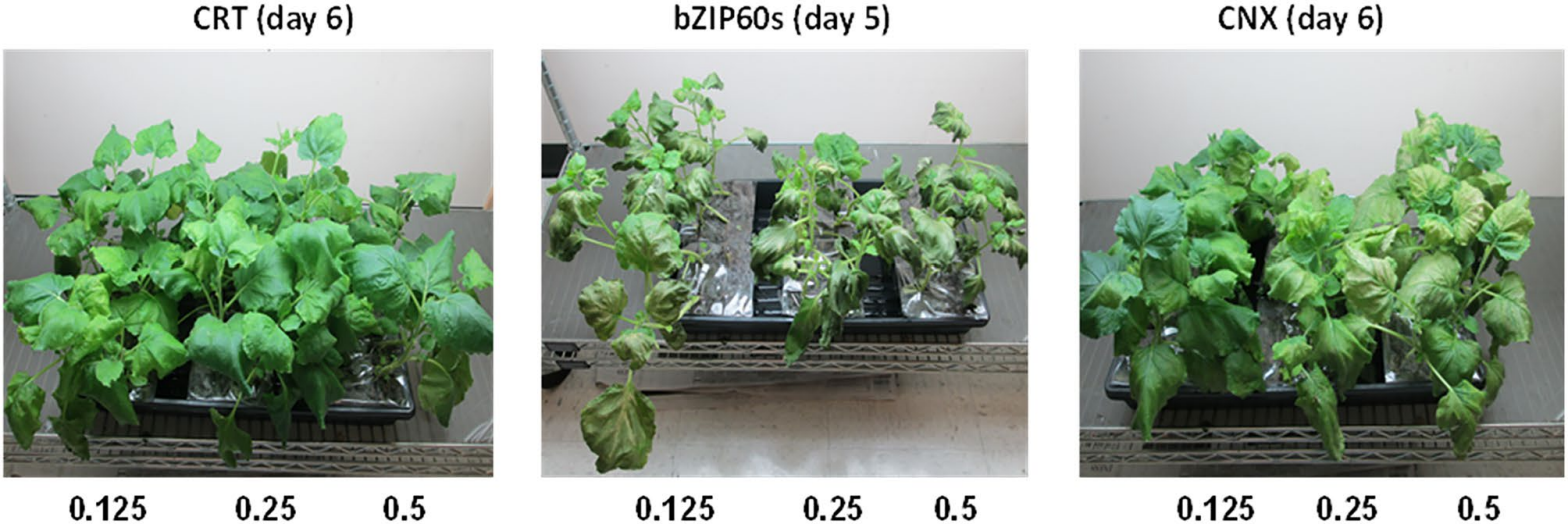
Assessment of toxicity of the plant master regulator bZIP60-s and the CRT and CNX chaperones in *N. benthamiana* leaves. Infiltrations were performed at different chaperone OD600 as indicated.

To assess whether increase in individual plant chaperone concentrations in plant cells would be detrimental to the leaves, plants were initially infiltrated with individual genes for two master regulators and each of 5 plant chaperones at OD600s of 0.125, 0.25 and 0.5. Expression of bZIP28-t, BiP_4, Erp57_13, CRT and PPI had no effect on the leave appearance at any ratio, similar to CRT (Fig. 3, left), while activated bZIP60-s was exceedingly toxic at all dosages tested OD600s (center) and CNX caused toxic signs at OD600s higher than 0.125 (right).

### Effect of chaperone genes on leaf health and levels of SOSIP expression in *N. benthamiana*

**Figure 4:**
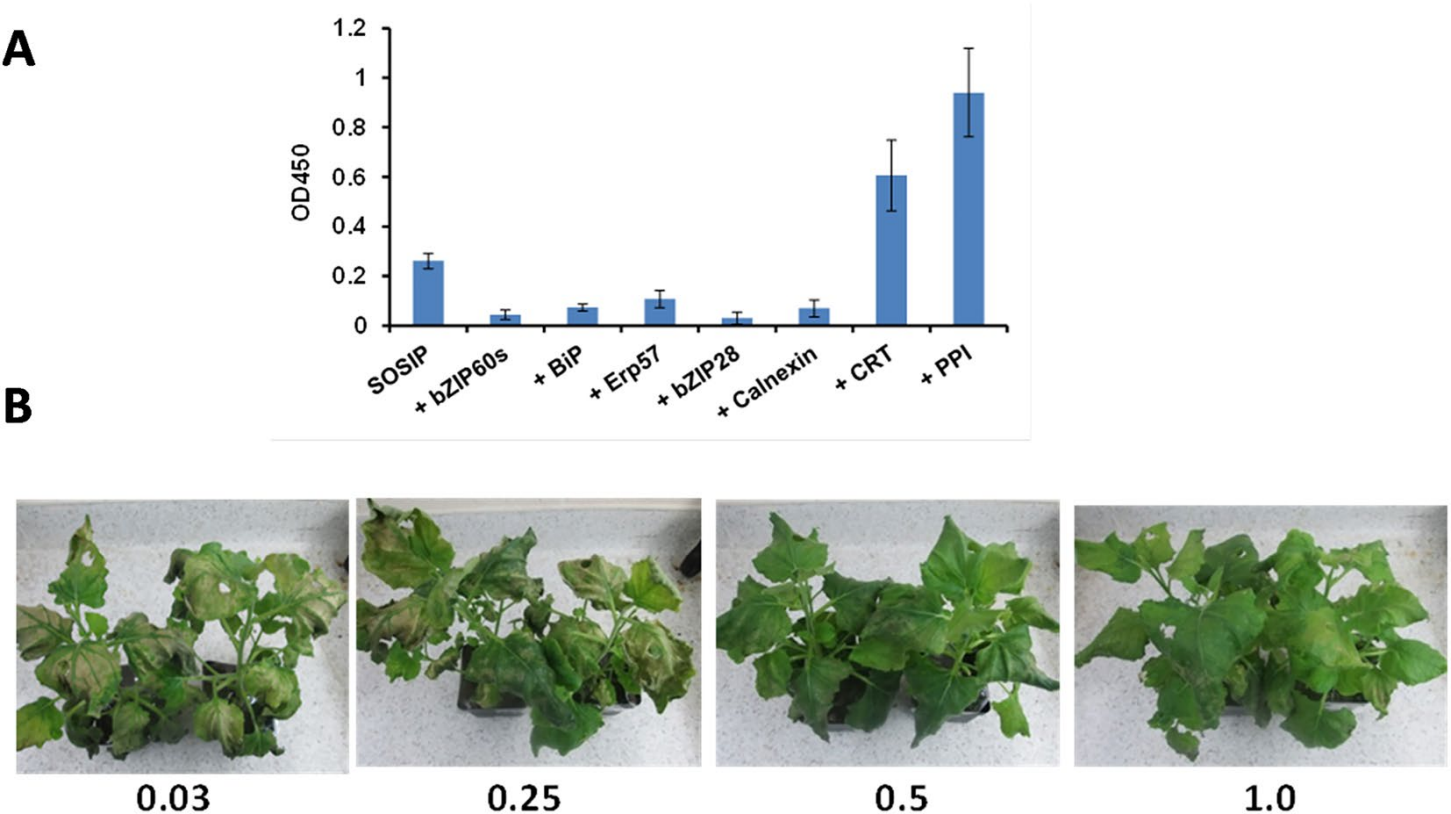
Effect of ER chaperones on expression in *N. benthamiala* and leaf pathology following co-infiltration of CH505 SOSIP chaperone genes at 10 dpi. (**A**). Enhancement of expression of the CH505 gene with individual chaperone genes. Leaf extracts were diluted sixfold. Chaperone genes were all infiltrated at OD600 = 0.2 except for Nb_bZIP60s gene, which was used at OD600 = 0.015. (**B**) Phenotypic screening of plants co-infiltrated with CH505 SOSIP and PPI at different OD600s.

Having assessed the impact on leaf health of the individual host cell factors alone at different OD600s, each was then co-transfected at a non-toxic OD600 with the CH505 SOSIP gene to assess whether they could facilitate correct folding of the HIV SOSIP trimer and increase the expression levels in the *N.b/p19* system. To achieve this, plants were co-infiltrated with genes for CH505 SOSIP along with those for the 7 host cell factors at OD600s ranging from 0.03 to 1.0. ELISA results using leaf extracts harvested at day 10 indicate that two of the seven chaperones resulted in significantly increased SOSIP expression (Fig. 4A). Thus, co-expression of PPI at the initial conditions used (OD600 0.2), resulted in up to fivefold increase in CH505 SOSIP expression while CRT increased levels up to threefold effect. The remaining three chaperones actually reduced SOSIP expression levels although no alteration in leaf appearance was observed. Figure 4B indicates plants remained healthy following infiltration with the PPI gene at an OD600 at 0.5 and 1.0 but not at lower OD600s (0.03, 0.06, 0.125 and 0.25). Similarly, CRT was optimal at 0.5 and above (not shown). It should be noted that co-transfection of leaves with the BiP_4 gene, prevented wilting and browning as a result of CH505 SOSIP toxicity. However, co-infiltration of the activated bZIP60s master regulator gene overrode the BiP_4 “effect” and resulted in toxicity again with severe necrosis of the leaves.

### Effect of combinations of chaperones on leaf health and levels of expression

**Figure 5:**
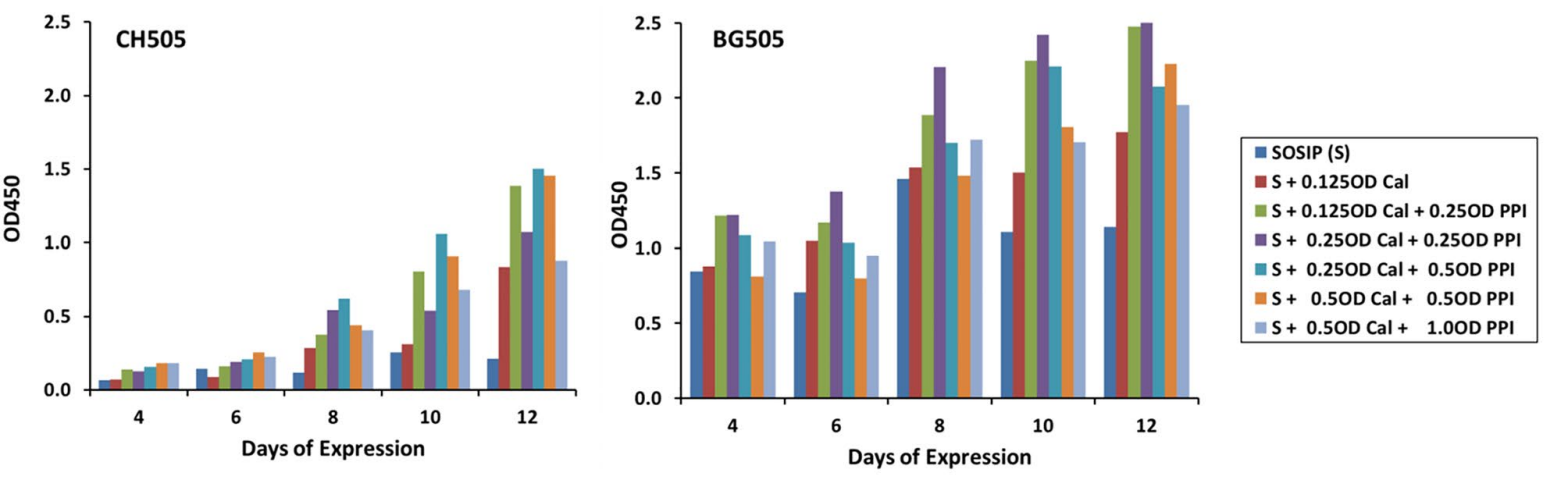
Phenotypic screening for leaf toxicity following infiltration of CH505 and BG505 SOSIP in the presence of PPI and PPI + CRT. CH505 (left) and BG505 (right) were infiltrated at OD600 = 0.35. In the absence of chaperones, the ELISA OD450 for CH505 is typically 0.1–0.3 and for the BG505 0.5–1.0.

Since PPI and CRT were the only chaperones shown to increase expression alone, it was important to examine possible additive or synergistic effects between them e.g. due to increased formation of protein clusters/foldase complexes. As a proof-of-concept study, the ability of PPI + CRT chaperones to prevent/delay leaf pathology and enhance expression was assessed using the plant-derived CH505 and scBG505 SOSIPs at different chaperone OD600 ratios. Figure 5 indicates that OD600 CRT:PPI combinations of 0.125:0.25, 0.25:0.25 and 0.25:0.5 were usually optimal in terms of ELISA OD450 levels and differed according to the SOSIP used. In this context, the BG505 SOSIP alone (right) was always expressed at higher levels in plants than CH505 in the absence of chaperones while the chaperone-mediated fold-increase in CH505 and CH848 was accordingly higher than that observed with the BG505 SOSIP. Leaf health was also greatly improved in all combinations of the chaperones. It should be noted that although chaperone-mediated expression levels increased with time, leaf pathology was again observed at 10–12 dpi; presumably due to the increased SOSIP reaching a threshold at the later time points. For some combinations this resulted in reduced yields in terms of mg/kg.

### Increased expression using SOSIP gene with ER-retrieval signal (KDEL)

**Figure 6:**
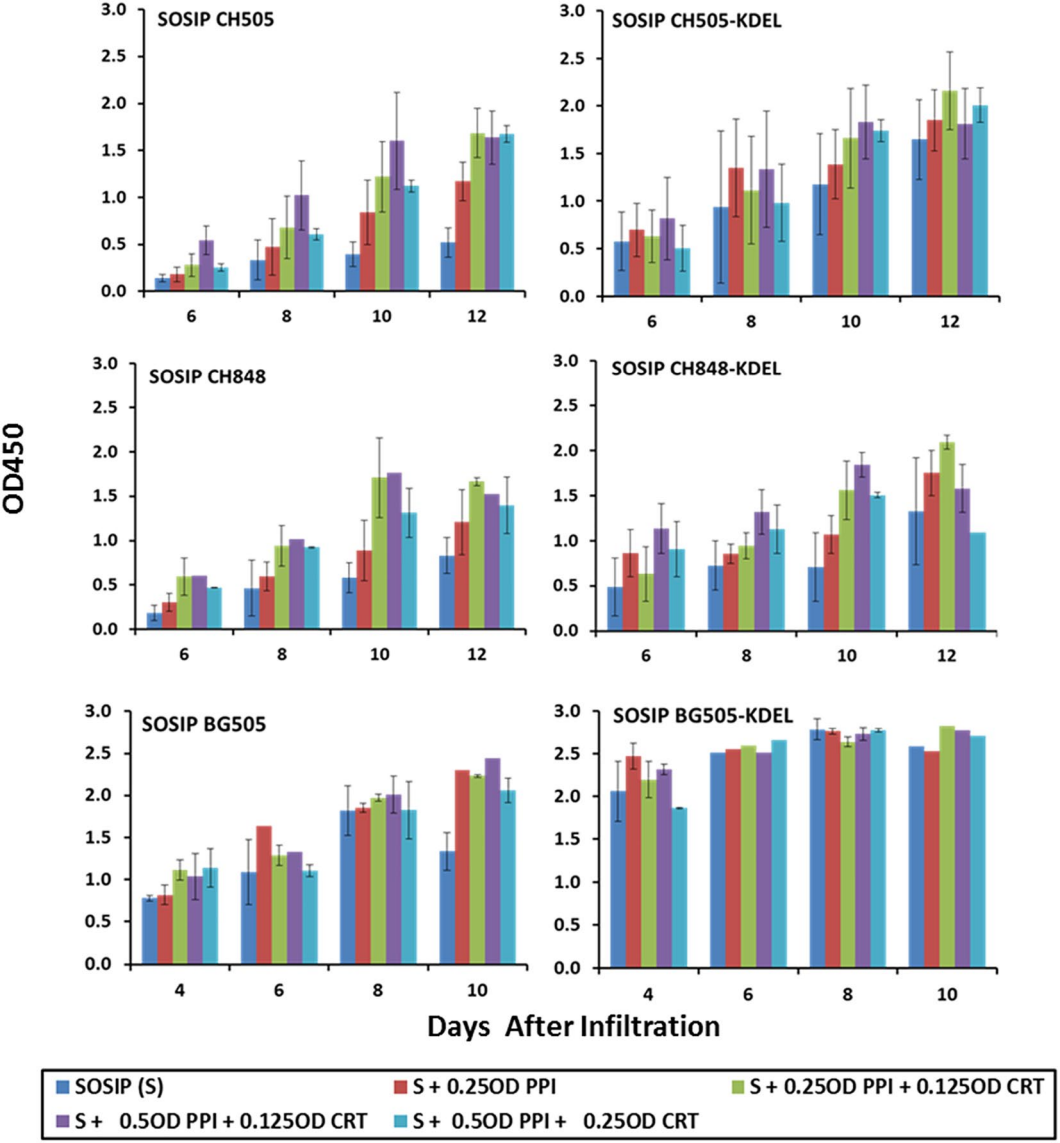
Chaperone mediated enhancement of KDEL forms of SOSIPs. Expression levels at different days after infiltration of non-KDEL and KDEL forms of CH505 (**A**), CH848 (**B**) and BG505 (**C**) were compared in the presence of different ratios of CRT and PPI chaperones. Based on ELISA titrations using purified BG505 SOSIP, the level of expression of plant-derived SOSIP Envelopes in several infiltrations was 30–60 mg/kg for BG505 and 10–40 mg/kg for the CH505 and CH848. It should be noted that 1/12 dilutions of extract were used for the CH505 and CH848 SOSIPS and BG505-KDELresults reached saturation by ~ day 6 at 1/24 dilutions.

Based on the observed enhancement of expression by CRT, an ER chaperone, deliberate prolonged retention in the ER of SOSIP Env glycoproteins and the potential for recycling through the UGGTI, would likely lead to higher increases in levels of correctly folded proteins and further enhancement of expression levels. To examine this possibility, KDEL forms of the SOSIP Env genes were generated and co-infiltrated with the CRT and PPI chaperones to assess the impact of an ER-retention signal on SOSIP accumulation at OD600 = 0.35. Figure 6 compares the PPI + CRT-enhanced accumulation using the KDEL forms of CH505 and BG505 SOSIP Env and a third mutant CH848 SOSIP Env and indicates (i) increased expression of SOSIP alone and further increases with chaperones at most time points compared to the non-KDEL forms (ii) earlier increased expression at days 6 to 8 with both CH505 and CH848-KDEL SOSIP and days 4 to 6 with the BG505 SOSIP. This is important because healthy leaves at this time yield a larger available biomass and can be more efficiently extracted. The CRT-mediated increases are more evident with KDEL forms of CH505 and CH848 at later time points and in some cases, PPI alone accounted for more of the observed enhancement with the KDEL forms, especially BG505-KDEL.

### Chaperone PPI and CRT genes inhibit SOSIP expression in *N. tabacum*

**Figure 7:**
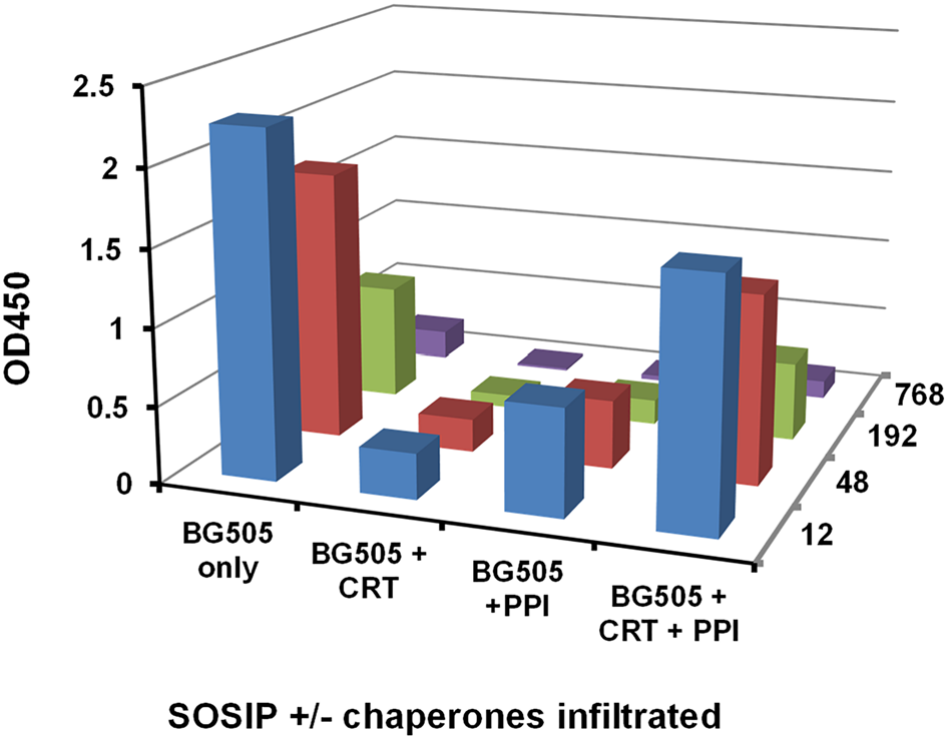
Expression levels of scBG505 SOSIP in *N.tabacum* extracts as measured by ELISA. Plants were infiltrated with genes for the scBG505 SOSIP and different combinations of CRT and PPI genes and harvested at day 8. Dilutions from 1/12 to 1/768 are shown for each group. Expression of scBG505 SOSIP alone in *N. tabacum* reached > 30 mg/kg.

As noted earlier, unlike the *N.b/p19* system, expression of SOSIP Env in *N. tabacum* in several experiments did not result in leaf browning or death at OD600s as high as 1.0. To examine whether ER chaperones would enhance expression, *N. tabacum* plants were infiltrated with the scBG505 SOSIP gene alone (OD 0.35) or together with PPI, CRT or PPI + CRT genes and harvested at day 8. In contrast to the high expression (equivalent to > 30 mg/kg*)* obtained in the absence of chaperones, expression levels were significantly reduced by chaperones in the order CRT > PPI > CRT + PPI (Fig. 7); indicating the different susceptibility to ER stress and the finely regulated control exerted by chaperones levels in a different but closely related *Nicotiana* plant species. Experiments in which leaves were harvested at days 6 and 12 showed similar profiles.

## Discussion

In contrast to small single-domain proteins which may spontaneously acquire their native folding with high efficiency immediately upon translation, larger complex proteins fold more slowly requiring the intervention of cellular factors such as molecular chaperones to assist and optimize the folding process and successful synthesis^45^. In this context, a growing number of diseases are now known to be associated with protein folding defects involving difficult-to-fold multi-domain proteins^54^. The large HIV Env glycoprotein trimer, which includes numerous disulfide bonds, proteolytic cleavage and extensive glycosylation typifies the challenges associated with misfolding and stress, especially in plants, where SOSIP production leads to leaf browning at 4–6 dpi and subsequent cell death. The association between reduced leaf pathology and enhanced expression in Env-infiltrated plants enabled a more rapid and simple evaluation of those conditions required to demonstrate chaperone-mediated effects. Three different SOSIP trimers, scBG505, CH505 and CH848*,* with known UCA binding and potential as germline-targeting immunogens, were selected to demonstrate chaperone-assisted expression in plants; the latter determined by ELISA using PGT145-coated plates to specifically recognize and capture trimer quarternary structures in plant extracts.

Initially, genes for 7 plant host cell factors were singly transfected at different dosages, i.e. Agro-OD600 ranging from 0.015 to 0.5, using the *N.b/p19* plant system to assess their toxicity and the optimal OD600 for subsequent co-infiltration with SOSIP genes. The effect of increasing the levels of these individual factors in plants cells differed widely, with the activated bZIP60s master-regulator being highly toxic even at OD600 as low as 0.015, indicating that overexpression of bZIP60s drives the UPR from a pro-survival to a cell death response, and thus is counterproductive in terms of alleviating the ER stress response caused by SOSIP overexpression. By contrast, the master regulator bZIP28t and all chaperones by themselves, except for CNX, did not cause leaf browning up to ODs of 0.5. Interestingly, the overriding of the “BiP effect” by bZIP60s shows mitigating Ire1 pathway activation appears more advantageous than (over-)activating the unfolded protein response.

Importantly, when co-transfected with any of the three SOSIP Env genes, only two of the 7 factors studied, PPI and CRT, both reduced/delayed cellular toxicity and enhanced expression by 3- to 15-fold depending on the time of harvest and the OD600 combinations tested. In this context, co-infiltration of the PPI gene with the WT SOSIP genes played a more substantial role in the observed increases, while the additive effects contributed by CRT appeared to depend on the form of the SOSIP and PPI:CRT OD600s, typically being optimal at 0.125:0.25, 0.25:0.25 and 0.25:0.5. A 12-fold enhanced expression of the HIV CAP256 gp140 SOSIP.664 glycoprotein, as a result of co-infiltration with the human CRT gene determined by gel densitometry and western blotting has been recently reported^35^.

Many host regulators and chaperones reside within the lumen of the ER to maintain nascent glycoproteins in a soluble and folding-competent state; facilitating transition into the Golgi and beyond. The accumulation of misfolded glycoproteins may impose ER stress and targeting to the ERAD^37^ with subsequent reduced/poor expression. This is rapidly evident visually in *N. benthamiana* plants following transfection with recombinant SOSIP Env genes by the resulting pathology observed in the infiltrated leaves. The finding that the two plant chaperones PPI and CRT, crucial for correct folding of glycoproteins in the ER, both overcame leaf pathology and enhanced expression, highlights the role misfolding of the HIV SOSIP Envs plays in their toxicity and the associated reduced accumulation. It should be noted however, that several factors did reduce *N. benthamiana* leaf browning but without any increase in expression, indicating their role in mitigating ER stress induced by transgene overexpression of leaves, other than those involved in SOSIP folding mechanisms.

While CRT is a soluble luminal homolog of the membrane-anchored CNX chaperone, and both bind to monoglucosylated *N*-glycans on recently synthesized nascent proteins, only CRT increased SOSIP Env expression; perhaps associated in part with the observed toxicity of CNX at OD600 > 0.125 (Fig. 3). Thus, in WT *N. benthamiana* plants, the CRT component of the CNX/CRT cycle surveys the folding status of soluble SOSIP glycoproteins in the ER and facilitates export to the Golgi of only the relatively low levels of proteins with native folding, while in the CRT-infiltrated plants, higher numbers of “almost native” folded glycoproteins are able to reenter the cycle and now attain correct conformation^45^. In this context, SOSIP expression levels alone were all increased when linked to KDEL. In addition, the larger CRT contribution to the PPI + CRT enhanced expression were observed with KDEL-tagged SOSIPS in later harvests; perhaps consistent with additional recycling in the CNX/CRT network as a result of retention/retrieval in the ER. This is most evident in terms of the ~ tenfold increase in yield 6–8 dpi following co-transfection of the CH848-KDEL and CH505 SOSIP genes with PPI and CRT chaperone genes compared to the non-KDEL forms. Currently, it is not clear whether the increases are due to the KDEL tag and the chaperones being additive or whether the “chaperone effect” reflects increased folding efficiency while the “KDEL effect” may provide a more favorable cellular compartment thereby reducing the rate of degradation of the folded protein.

By contrast, all three subfamilies of PPIase (peptidyl-prolyl cis–trans isomerase), cytophilin (CyP), FK506-binding protein (FKBP) and parvulin (Pvn)^55–57^ accelerate the folding process and increase the yield of molecules that reach the native state, by accelerating the rate-limiting isomerization of Xaa-Pro peptide bonds. Proline often plays a critical rate limiting role in protein folding because of the energy barrier of the cis and trans isomerization and the large impact on protein conformation. It is noteworthy that PPI accounted for most of the chaperone-mediated increases with the BG505 SOSIP and at early time points with the CH505 and CH848 SOSIPS.

*Agrobacterium*-mediated transient expression in plant leaves facilitates co-expression of several genes to produce complex hetero-oligomeric proteins as well as to manipulate the host cell. In addition to the SOSIP and chaperone genes, the human furin gene was co-infiltrated into both *N. benthamiana* an*d N. tabacum* since the plant furin enzyme does not cleave HIV Env efficiently. Human furin has been successfully produced in plants^35,58^ and in the present studies was co-expressed with the CH505 and CH848 SOSIPS to provide proteolytic cleavage but was not necessary for the single chain cleavage independent BG505. A fourth gene for p19, an plant viral inhibitor of silencing known to significantly increase expression of plant antibodies and enzymes^26,28^ was shown to enhance SOSIP levels in *N. benthamiana* but was not effective in *N. tabacum* plants*.*

In terms of SOSIP expression in *N. tabacum*, unexpected findings highlighted the complex role of ER chaperones and the need for fine-tuning the biosynthesis machinery in closely related species. Thus, in contrast to the marked toxicity observed in *N. benthamiana* plants, significant BG505 SOSIP expression levels of > 30 mg/kg of leaf biomass, was achieved in *N. tabacum,* with no pathology in several experiments. Indeed the presence of the *N. benthamiana* CRT and PPI significantly reduced SOSIP expression (Fig. 7), highlighting the importance of the genetic background as well as yet unknown functional differences between evolutionary conserved chaperone genes^35^.

Currently, the impact of CRT and PPI co-expression on the N-glycan profile of plant-derived SOSIP Env is unknown but potentially important in terms of immunogenicity, since individual glycans on candidate vaccine SOSIP trimers may both comprise the epitopes for bnAbs and also shield nearby sites that might otherwise dominate immune responses. In a previous plant study, an HIV 89.6P gp140DCFI Env was shown to be predominantly oligo-mannose (79%)^26^, especially KDEL forms which were 100% OMT. In this context, recombinant plant Env more closely resemble the glycosylation profiles of the native virion gp120 comprising the Env trimers^59,60,63^. Thus, soluble well-formed trimer patches of glycans on gp120 contain mainly high mannose-type glycans, while complex glycans are enriched only in the gp41 region. Man5-enriched N-glycans on gp120 of the trimeric CH505 T/M virions have recently been shown to provide additional synergy for neutralization by the CH235 unmutated common ancestor (UCA)^61^. It should also be noted that the plant specific β-1,2-xylose and core α-1,3-fucose can now be easily eliminated^62^ from plant-produced vaccine glycoproteins.

In summary, the versatility, low entry cost and high speed of plant-based transient gene expression systems are highly advantageous for testing and developing novel expression and manufacturing strategies for complex targets such as HIV Envs. Many findings will be translatable to other complex glycoproteins and production hosts, e.g. SARS-CoV2 spike proteins and CHO cells. The ability to mix and match the *Agrobacteria* as well as to adjust the SOSIP and chaperone gene "dosage" by varying the ratios of the OD600 proved to be important in determining limitations and achieving optimized expression and may not be achievable with chaperone-transgenic cell lines.

## Methods

All plant experiments have been carried out in accordance with relevant institutional, national and international guidelines and legislation.

### Cloning of plant genes for co-expression

The genes for two master regulators and five chaperones from *N. benthamiana* were blasted and retrieved from the *Nicotiana benthamiana* Genome Database with sequence analysis (intron/exon, pseudo-gene yes/no, homologs) through the Solgenomics webpage: https://www.solgenomics.net/tools/blast/?db_id=266, Table 1 is the genome region of each chaperone:

**Table 1:** Genome location of *Nicotiana benthamiana* master regulators and chaperones.

Chaperone	*N. benthamiana* genome region
b.ZIP60	Niben101Scf24096:86678.0.89465 (− strand)
bZIP28	Niben101Scf00077:854681.0.858431 (− strand)
BiP, isoform 4	Niben101Scf08590:10860.0.15217 (+ strand)
PDI (Erp57)	Niben101Scf10528:184598.0.190939 (− strand)
Calnexin (CNX)	Niben101Scf03777:164368.0.168487 (+ strand)
Calreticulin (CRT)	Niben101Scf00466:539066.0.543802 (− strand)
PPI-B	Niben101Scf04126:114012.0.121787 (+ strand)

Three SOSIP Envs were studied, with coding sequences for CH505TF.6R.SOSIP.664.v4.1 and CH848.3.D0949.10.17CHIM.6R.SOSIP.664V4.1 being kindly provided by Dr. Barton Haynes, Duke University, and the single chain BG505 SOSIP gp140 sequence and a 15 aa Gly-Ser linker being based on^14^. All SOSIP genes and those for seven ER cell factors were synthesized by SynBio (NJ). Plasmid pMD-furin, which contains human furin (NP_001276752.1) was purchased from Sino Biological (Chesterbrook, PA). Synthetic genes were inserted into a pTRAk expression vector at EcoRI and BamHI sites using standard approaches. Positive plasmids, verified by DNA sequencing, were individually electroporated into *A. tumefaciens* strain GV3101 (pMP90RK) and selected on YEP agar plates containing 50 μg/mL rifampicin, 50 μg/mL carbenicillin and 30 μg/mL kanamycin (Sigma-Aldrich). Positive transformants were verified by PCR using vector and insert specific primer pairs. The pTRAk-TBSV containing the tomato bushy stunt virus p19 inhibitor of silencing (AJ288917) has been described previously^26^. For all three SOSIP Env, two constructs were made with and without a C-terminal SEKDEL tag.

### *Agrobacterium*-mediated transient gene expression of HIV SOSIP Env

Recombinant *A. tumefaciens* glycerol stocks were grown in YEP supplemented with 50 μg/mL rifampicin, 50 μg/mL carbenicillin and 30 μg/mL kanamycin, in a temperature controlled shaker (27 °C/150 rpm) over several days to saturation measured by *Agro-bacterium* optical density. The culture was pelleted and resuspended in MS medium containing 20 μM acetosyringone and 20 g/L of sucrose to a working OD (usually 0.5 or less for infiltration of 5–6 week old plants). The transfected “gene dosage” was estimated using OD600. After infiltration, plants were placed at 20 °C, with a 16 h/ 8 h, light/dark cycle and leaves harvested 4 to 12 days later while also being monitored and photographed for leaf pathology.

Plant chaperone genes were initially infiltrated individually into *N. benthamiana* plants at various ODs: 0.125, 0.25 and 0.5. Later, plants were co-transfected along with the SOSIP (OD60 0.2–0.35), furin and p19 genes. The gene ratio of SOSIP: furin: p19 was usually 6:2:1, but the gene ratio was refined with the addition of the chaperone genes. For Infiltration of *N. tabacum*, *s*ix week old tobacco plants (cultivar B21) were infiltrated with SOSIP BG505 alone (OD600 = 0.35) or in combination with chaperones CRT and PPI. Plants were examined for pathology daily and photographed. Samples were harvested at different days and extracted immediately or frozen at − 20 °C until purification.

### Screening of plants

For initial screening, six leaf discs (~ 10 mg each) were collected from different positions on the transfected leaves (Fig. 3), ground in 200 ul PBS, centrifuged, frozen at − 20 °C and tested for the presence of Env by ELISA or dot blot or Western using CHO- and/or plant-derived HIV bnAbs (not shown).

### Affinity purification of BG505 SOSIP

Protein A-purified (Genscript, L00433) plant-derived 2G12 HIV antibody^25^ was conjugated to CNBr-activated Sepharose™ 4B resin (GE Healthcare, 17-0430-01) in the presence of 0.1 M d-fructose following manufacturer’s instructions. The column was equilibrated in PBS before use. Infiltrated leaves were ground using a Vitamix blender in PBS buffer, pH 7.5, containing 5 mM magnesium chloride, 4 mM dithiothreitol, 5 mM sodium metabisulfite, 10% sucrose, and 1% polyvinylpyrrolidone (MW40,000) (buffer:leaf mass = 5:1). Extract was passed through Miracloth and centrifuged at 18,500×*g* at 4 °C for 15 min. The supernatant was collected and the pH increased to 7.5 with sodium hydroxide. Chitosan (1% mass/volume) was dissolved in 1% acetic acid and added to the leaf extract at a final concentration of 0.02% (mass/volume) and stirred at 4 °C for 30 min and then left without stirring for another 30 min at 4 °C. After centrifugation at 18,500×*g* at 4 °C for 15 min, the extract was stirred with DEAE Sephadex A-25 (1% mass/ volume) for 1.25 h at 4 °C and then left without stirring for an additional 10 min at 4 °C. The extract was filtered through a 0.45 µm PES vacuum filter, adjusted to pH 7.5 and loaded onto a 2G12-Sepharose column. Columns were washed initially with PBS and then a buffer containing 20 mM tris, pH 8, and 500 mM NaCl after which SOSIP Env was eluted with a solution of 3 M magnesium chloride and 50 mM tris. Fractions containing protein, determined by the absorbance at 280 nm, were pooled and concentrated using an Amicon ultra-15 centrifugal filter unit (30 kDa MWCO) and the solution dialyzed in 6–8 kDa MWCO tubing (Spectrapor) in 20 mM tris, pH 8, and 75 mM NaCl. Purified BG505 SOSIP was frozen at − 20 °C.

### ELISA

Sandwich ELISAs were used at RT to measure the expression level of trimeric HIV SOSIP Env in both leaf extracts and as purified proteins. 96-well MaxiSorp plates (Nunc) were coated for 2 h in 100 ul of PBS containing 400 ng of plant-derived PGT145, purified by protein A and MEP HyperCel (Pall, 12035-208) chromatography. After blocking with 5% (w/v) nonfat dry milk in PBST, wells were washed 3 times with PBST, incubated with plant samples at different dilutions or concentrations for 2 h. Following 3 washes, wells were incubated with 400 ng of 2G12-KDEL-biotin for 1.5 h, washed and incubated with 10 ng of streptavidin (HRP) (AbCam, ab7403), washed 5 times, and developed with KPL SureBlue Reserve TMB Microwell Peroxidase Substrate (1-Component) (SeraCare, 5120-0083) for 6 min. The reactions were stopped with 0.5 M H2SO4, and absorbance at 450 nm measured using the SPECTRA max PLUS plate reader (Molecular Devices). Wells without leaf extracts and unfiltrated leaf extracts were used as controls. Purified CHO-derived BG505 SOSIP.664-His, a generous gift from Dr. John Moore, or plant-derived 3A4-CH505 SOSIP were used as a positive control.

### Ethical statement

This article does not contain any studies with human participants or animals performed by any of the authors.

## References

[1] Scheid JF (2009). Broad diversity of neutralizing antibodies isolated from memory B cells in HIV-infected individuals. Nature.

[2] Walker LM (2009). Broad and potent neutralizing antibodies from an African donor reveal a new HIV-1 vaccine target. Science.

[3] Moldt B (2012). Highly potent HIV-specific antibody neutralization in vitro translates into effective protection against mucosal SHIV challenge in vivo. Proc. Natl. Acad. Sci. USA.

[4] Hessell AJ (2009). Effective low-titer antibody protection against low-dose repeated mucosal SHIV challenge in macaques. Nat. Med..

[5] Rosenberg YJ (2016). Protection against SHIV challenge by subcutaneous administration of the plant-derived PGT121 broadly neutralizing antibody in Macaques. PLoS ONE.

[6] Barouch DH (2013). Therapeutic efficacy of potent neutralizing HIV-1-specific monoclonal antibodies in SHIV-infected rhesus monkeys. Nature.

[7] Klein F (2012). HIV therapy by a combination of broadly neutralizing antibodies in humanized mice. Nature.

[8] Caskey M (2015). Viraemia suppressed in HIV-1-infected humans by broadly neutralizing antibody 3BNC117. Nature.

[9] Eroshkin AM (2014). bNAber: Database of broadly neutralizing HIV antibodies. Nucleic Acid Res..

[10] Burton DR, Hangartner L (2016). Broadly neutralizing antibodies to HIV and their role in vaccine design. Annu. Rev. Immunol..

[11] Karuna ST, Lawrence Corey L (2020). Broadly neutralizing antibodies for HIV prevention. Annu. Rev. Med..

[12] Julian JP (2013). Crystal structure of a soluble cleaved HIV-1 envelope trimer. Science..

[13] Dubrovskaya V (2019). Vaccination with glycan-modified HIV NFL envelope trimer-liposomes elicits broadly neutralizing antibodies to multiple sites of vulnerability. Immunity..

[14] Georgiev IS (2015). Single-chain soluble BG505 SOSIP gp140 trimers as structural and antigenic mimics of mature closed HIV-1 *Env*. J. Virol..

[15] Sanders RW (2013). A next-generation cleaved, soluble HIV-1 Env trimer, BG505 SOSIP.664 gp140, expresses multiple epitopes for broadly neutralizing but not non-neutralizing antibodies. PLoS Pathog..

[16] Dey AK (2018). cGMP production and analysis of BG505 SOSIP.664, an extensively glycosylated, trimeric HIV-1 envelope glycoprotein vaccine candidate. Biotechnol. Bioeng..

[17] Saunders KO (2017). Vaccine induction of heterologous tier 2 HIV-1 neutralizing antibodies in animal models. Cell Rep..

[18] Torrents de la Peña A (2018). Immunogenicity in rabbits of HIV-1 SOSIP trimers from clades A, B, and C, given individually, sequentially, or in combination. J. Virol..

[19] Bale S (2018). Cleavage-independent HIV-1 trimers from CHO cell lines elicit robust autologous tier 2 neutralizing antibodies. Front. Immunol..

[20] Schorcht A (2020). Neutralizing antibody responses induced by HIV-1 envelope glyco protein SOSIP trimers derived from elite neutralizers. J Virol..

[21] Sullivan JT (2017). High-throughput protein engineering improves the antigenicity and stability of soluble HIV-1 envelope glycoprotein SOSIP trimers. J. Virol..

[22] Pauthner MG (2019). Vaccine-induced protection from homologous Tier 2 SHIV challenge in nonhuman primates depends on serum-neutralizing antibody titers. Immunity.

[23] Stoger E, Fischer R, Moloney M, Ma JK (2014). Plant molecular pharming for the treatment of chronic and infectious diseases. Annu. Rev. Plant Biol..

[24] Sack M, Hofbauer A, Fischer R, Stoger E (2015). The increasing value of plant-made proteins. Curr. Opin. Biotechnol..

[25] Buyel JF, Twyman RM, Fischer R (2015). Extraction and downstream processing of plant-derived recombinant proteins. Biotechnol. Adv..

[26] Rosenberg YJ (2013). Rapid high-level production of functional HIV broadly neutralizing monoclonal antibodies in transient plant expression systems. PLoS ONE.

[27] Menzel S (2016). Optimized blanching reduces the host cell protein content and substantially enhances the recovery and stability of two plant-derived malaria vaccine candidates. Front. Plant Sci..

[28] Rosenberg YJ (2015). A highly stable minimally processed plant-derived recombinant acetylcholinesterase for nerve agent detection in adverse conditions. Sci. Rep..

[29] The PREVAIL II Writing Group, for the Multi-National PREVAIL II Study Team (2016). A randomized, controlled trial of ZMapp for Ebola virus infection. N. Engl. J. Med..

[30] Laffan AM (2011). Lactoferrin for the prevention of post-antibiotic diarrhoea. J. Health Popul. Nutr..

[31] Taylor, N. P. *For FierceBiotech. Medicago, GSK Post Positive Midphase Data on Plant-Derived COVID-19 Vaccine*. https://fiercebiotech.com/biotech/medicago-gsk-post-positive-midphase-data-plant-derived-covid-19-vaccine (2021)

[32] Behrens A-J (2016). Composition and antigenic effects of individual glycan sites of a trimeric HIV-1 envelope glycoprotein. Cell Rep..

[33] Go EP (2017). Glycosylation benchmark profile for HIV-1 envelope glycoprotein production based on eleven Env trimers. J. Virol..

[34] Margolin E (2019). Production and immunogenicity of soluble plant-produced HIV-1 subtype C envelope gp140 immunogens. Front Plant Sci..

[35] Margolin E (2020). Co- expression of human calreticulin significantly improves the production of HIV gp140 and other viral glycoproteins in plants. Plant Biotechnol. J..

[36] Pera FF (2015). Engineering and expression of a human rotavirus candidate vaccine in *Nicotiana benthamiana*. Virol. J..

[37] Hamorsky KT (2015). N-glycosylation of cholera toxin B subunit in Nicotiana benthamiana: Impacts on host stress response, production yield and vaccine potential. Sci. Rep..

[38] Qi L, Tsai B, Arvan P (2017). New Insights into the physiological role of endoplasmic reticulum-associated degradation. Rev. Spec. Ser. Mitochondria..

[39] Angelos E, Ruberti C, Kim S-J, Brandizzi F (2017). Maintaining the factory: The roles of the unfolded protein response in cellular homeostasis in plants. Plant J..

[40] Borsa M (2015). HIV infection and antiretroviral therapy lead to unfolded protein respo. Virol. J..

[41] Shah A, Vaidya NK, Bhat HK, Kumar A (2016). HIV-1 gp120 induces type-1 programmed cell death through ER stress employing IRE1α, JNK and AP-1 pathway. Sci. Rep..

[42] Zhang L, Wang A (2012). Virus-induced ER stress and the unfolded protein response. Front. Plant Sci..

[43] Ye C, Dickman MB, Whitham SA, Payton M, Verchot J (2011). The unfolded protein response is triggered by a plant viral movement protein. Plant Physiol..

[44] Lamriben L, Graham JB, Adams BM, Hebert DN (2016). N-glycan-based ER molecular chaperone and protein quality control system: The calnexin binding cycle. Traffic.

[45] Ferris SP, Kodali VK, Kaufman RJ (2014). Glycoprotein folding and quality-control mechanisms protein-folding diseases. Dis. Model Mech..

[46] Gardner BM, Pincus D, Gotthardt K, Gallagher C, Walter P (2013). Endoplasmic reticulum stress sensing in the unfolded protein response. Cold Spring Harb. Perspect. Biol..

[47] Haverland NA, Fox HS, Ciborowski P (2014). Quantitative proteomics by SWATH-MS reveals altered expression of nucleic acid binding and regulatory proteins in HIV-1-infected macro- phages. J. Proteome Res..

[48] Scholz C (2005). Functional solubilization of aggregation-prone HIV envelope proteins by covalent fusion with chaperone modules. J. Mol. Biol..

[49] Pobre KFR, Poet GJ, Hendershot LM (2019). The endoplasmic reticulum (ER) chaperone BiP is a master regulator of ER functions: Getting by with a little help from ERdj friends. J. Biol. Chem..

[50] Snapp EL (2017). Structure and topology around the cleavage site regulate post-translational cleavage of the HIV-1 gp160 signal peptide. Elife..

[51] Liao H-X (2013). Co-evolution of a broadly neutralizing HIV-1 antibody and founder virus. Nature.

[52] Saunders KO (2019). Targeted selection of HIV-specific antibody mutations by engineering B cell maturation. Science.

[53] Lee JH (2017). A broadly neutralizing antibody targets the dynamic HIV envelope trimer apex via a long, rigidified, and anionic β-hairpin structure. Immunity.

[54] Yoshida H (2007). ER stress and diseases. FEBS J..

[55] Xu M, Meng W, Ma X (1995). PDI-, PPI- and chaperone-catalyzed refolding of recombinant human IL-2 and GM-CSF. Sci. China B..

[56] Lilie H, Lang K, Rudolph R, Buchner J (1993). Prolyl isomerases catalyze antibody folding in vitro. Protein Sci..

[57] Joseph AP, Srinivasan N, Alexandre G, Brevern DE (2012). Cis-trans peptide variations in structurally similar proteins. Amino Acids.

[58] Mamedov T (2019). Engineering, and production of functionally active human Furin in *N. benthamiana* plant: In vivo post-translational processing of target proteins by Furin in plants. PLoS ONE.

[59] Go EP (2017). Glycosylation benchmark profile for HIV-1 envelope glycoprotein production based on eleven Env trimers. J. Virol..

[60] Cao L (2018). Differential processing of HIV envelope glycans on the virus and soluble recom-binant trimer. Nat. Commun..

[61] LaBranche CC (2019). Neutralization-guided design of HIV-1 envelope trimers with high affinity for the unmutated common ancestor of CH235 lineage CD4bs broadly neutralizing antibodies. PLoS Pathog..

[62] Jansing J, Sack M, Augustine SM, Fischer R, Bortesi L (2019). CRISPR/Cas9-mediated knockout of six glycosyltransferase genes in *Nicotiana benthamiana* for the production of recombinant proteins lacking β-1,2-xylose and core α-1,3-fucose. Plant Biotechnol. J..

[63] Struwe WB (2018). Site-specific glycosylation of virion-derived HIV-1 Env is mimicked by a soluble trimer immunogen. Cell Rep..

